# Perturbations in Microbial Communities at Hydrothermal Vents of Panarea Island (Aeolian Islands, Italy)

**DOI:** 10.3390/biology14010086

**Published:** 2025-01-17

**Authors:** Annamaria Gallo, Fabio Sposito, Manfredi Longo, Gianluca Lazzaro, Cinzia Giuseppina Caruso, Sabina Morici, Sergio Scirè Scappuzzo, Slobodanka Radovic, Valeria Villanova, Luca Vecchioni, Marco Arculeo, Rosa Alduina

**Affiliations:** 1 Dipartimento Scienze e Tecnologie Biologiche, Chimiche e Farmaceutiche, University of Palermo, Viale delle Scienze, 90128 Palermo, Italy; annamaria.gallo01@unipa.it (A.G.); valeria.villanova@unipa.it (V.V.); luca.vecchioni@unipa.it (L.V.); marco.arculeo@unipa.it (M.A.); 2 Istituto Nazionale di Geofisica e Vulcanologia—Sezione di Palermo, Via Ugo La Malfa, 90146 Palermo, Italy; fabio.sposito@ingv.it (F.S.); manfredi.longo@ingv.it (M.L.); gianluca.lazzaro@ingv.it (G.L.); cinzia.caruso@ingv.it (C.G.C.); sabina.morici@ingv.it (S.M.); sergio.scire@ingv.it (S.S.S.); 3 IGA Technology Services, Via Linussio 51, 33100 Udine, Italy; sradovic@igatechnology.com; 4 NBFC National Biodiversity Future Center, Piazza Marina 61, 90133 Palermo, Italy

**Keywords:** 16S rRNA gene metabarcoding, hydrothermal vents, microbial community

## Abstract

Marine hydrothermal ecosystems represent extreme environments connected to submarine volcanic areas characterized by vents, having high temperatures and particular chemical compositions. The hydrothermal marine system of Panarea, located in one of the seven small islands belonging to the Aeolian Archipelago (southern Tyrrhenian Sea), is characterized by a range of vents exhibiting diverse physical and chemical conditions. We aimed to analyze the microbial community of a peculiar hot spring belonging to the Panarea hydrothermal field, known as “Black Point” (BP), in two separate sampling expeditions (May and August). Our results demonstrated that the chemical–physical variations within this hydrothermal vent, such as temperature fluctuations, mineral content, and hydrothermal fluid dynamics, play a role in shaping the structure and diversity of microbial communities. The differences between the two sampling expeditions suggest that seasonal changes, i.e., in temperature, pH, and redox potential (Eh), could drive microbial community shifts over time.

## Simple Summary

Marine hydrothermal ecosystems represent extreme environments connected to submarine volcanic areas characterized by vents, having high temperatures and particular chemical compositions. Microbial communities in hydrothermal vents play a crucial role in the functioning of these extreme environments and have significant ecological and scientific importance, with bacteria being the primary producers in the food chain and important for the cycling of essential elements such as carbon, sulfur, nitrogen, and metals. In addition, a deep investigation of the microbial communities in hydrothermal vents could offer a wealth of scientific knowledge, from an understanding of life in extreme conditions to potential applications in biotechnology and beyond.

## 1. Introduction

Submarine hydrothermal ecosystems represent extreme environments connected to underwater volcanic areas where numerous vents discharge hot fluids. Studying these ecosystems has been essential in understanding how organisms adapt to specific environments affected by the release of heated water. Indeed, the heated water that comes out of hydrothermal vents changes chemical and physical conditions, such as temperature, pH, electrical conductivity, and redox potential (Eh), in the surrounding environment [1,2,3].

In recent years, advancements in high-throughput genomic technologies, particularly metabarcoding using next-generation sequencing (NGS), have revolutionized the study of microbial communities. As an example, sulfur-oxidizing and hydrogen-oxidizing bacteria, as well as archaea, are well adapted to high to moderate temperatures and use oxygen or other electron acceptors to oxidize reduced sulfur compounds, which results in energy-producing (redox) processes. Bacteria employ the energy generated by these inorganic redox reactions for their biochemical and metabolic functions [4].

Since the discovery of hydrothermal vents, their unique chemical and physical properties have captured the scientific community’s attention. Over the past few decades, research has primarily focused on studying the microbial communities in these environments. In particular, studies on the variation in microbial presence between active and inactive vents, the succession patterns of different bacterial groups, and how these communities adapt to distinct chemical and physical conditions have been reported [5,6,7,8]. Additionally, microbial metabolic potential has been explored for different biotechnological applications [9,10,11].

**Figure 1:**
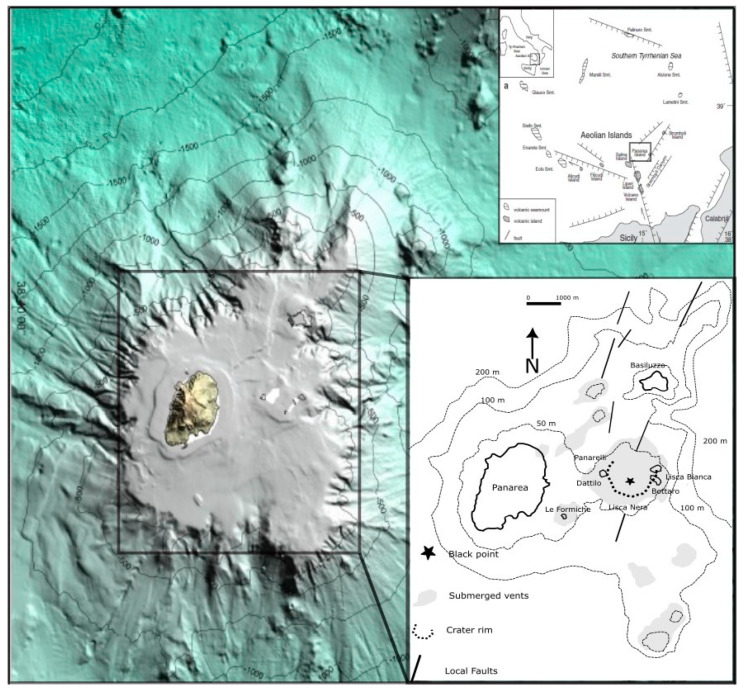
Morpho-bathymetric map of Panarea Island and relative hydrothermal field (modified after Longo et al. 2021 [26]). The black star marks the location of the studied site.

Panarea is the smallest island of the Aeolian Archipelago, located in the southern part of the Tyrrhenian Sea [12,13,14]. A submerged fumarolic field is located 2.5 km away from Panarea Island at a relatively shallow depth (<35 m b.s.l.), surrounded by five emerging reefs, named Dattilo, Bottaro, Lisca Bianca, Lisca Nera, and Panarelli, representing the remnants of an old volcanic center (Figure 1) [15,16,17]. In the late 1980s, researchers began a systematic investigation in the field of geochemistry, revealing the presence of a deep geothermal reservoir at temperatures ranging from 220 to 280 °C [18]. Panarea’s hydrothermal system has always shown intense dynamic variability [19]. In November 2002, a sudden deep magmatic input produced a huge gas blast on the seafloor [20]. The fluid fluxes released increased by two orders of magnitude and persisted for several months before the restoration of normal conditions [21,22,23,24]. After the crisis, several geo- and morpho-bathymetric surveys in the area highlighted the presence on the seafloor of hundreds of craters [22,25], indicating the highly dynamic nature of these systems, resembling the one identified in 2002. Moreover, another study on the behavior and composition of the fluids suggested a common source and a close correlation between the Panarea hydrothermal system and Stromboli volcanic activity [26]. Among the vents of the Panarea hydrothermal system, a site, referred to as Black Point (BP hereinafter), was discovered during a scuba diving investigation carried out after the explosive event on the 3rd of November 2002. BP is characterized by a brown-colored fluid enriched in iron (Fe^2+^), aluminum (Al^3+^), and manganese (Mn^2+^). Salt precipitation occurs when the hot mineral-rich fluids released by the vents rapidly mix with the cold seawater. As the fluids cool upon contact with the surrounding seawater, they become supersaturated with dissolved minerals. This supersaturation triggers the precipitation of insoluble metal oxy-hydroxides. The aggregation of these minerals results in dark-colored precipitates or coatings on surfaces next to the vents, giving them their characteristic black appearance.

The precipitation of Fe-Al-Mn oxy-hydroxides shapes the surrounding geological and ecological features [27,28,29]. These mineral deposits may accumulate over time, forming chimney-like structures, similarly to what happens in deep-ocean “black smoker” formation, or coating surrounding substrates with distinctive layers of minerals [30]. Additionally, the precipitated aggregates can serve as substrates for microbial colonization and play a role in geochemical cycling processes within hydrothermal ecosystems.

Despite the peculiarity of the Aeolian Archipelago and Black Point, as far as the authors know, previous reports have investigated the microbial component only of the shallow-water hydrothermal vents of this area [31,32,33,34,35,36,37]. In this study, for the first time, we characterized the microbial communities at the youngest hydrothermal vents of Panarea by combining the environmental metabarcoding of 16S rRNA gene amplicons with physicochemical features during two sampling expeditions (May and August 2022). This sampling strategy was designed to provide a detailed examination of the influence of vent modulation on the microbial communities of the hydrothermal water (HW), seawater (SW), and marine sediment (MS). This study aimed to understand the microbial communities in these extreme environments.

## 2. Materials and Methods

### 2.1. Study Site and Sampling Collection

The study site (23 m depth) is the so-called “Black Point”, a hot water spring just in the middle of the hydrothermal field. This vent emits hot fluid (138–140 °C, pH 2.3–2.8, Eh 150–230 mV) (see Table 1), which contains black sulfide and manganese incrustations (Figure 1) [38]. Sampling was carried out in two surveys in May and August 2022. Using sterile bottles, we collected 0.5 L of HW from the emission point and a 0.5 L SW sample near the emission point (5 m away). The exact point of emission of the hot spring was localized univocally using a thermometer, with the hole where the temperature reached its maximum value chosen as the collection point. Water samples from the source were collected using a sterile Teflon probe inserted into the source hole and connected to a syringe and Pyrex bottle via a three-way valve. The Pyrex sampling bottles were sterilized beforehand and filled with Millipore water to avoid any external contamination. Once at the BP site on the seabed, the sampling bottle was evacuated of Millipore water using air from the diving regulator. The bottle, equipped with two valves to allow the sample in and air out, was then connected to the three-way valve. The hydrothermal water samples were aspirated through the syringe and then pushed into the Pyrex bottle by turning the three-way valve, with this process repeated until the bottles were completely filled with hydrothermal water, avoiding any seawater contamination. Seawater samples near the seafloor, at a 5 m depth, and near the surface were collected by simply allowing the seawater to fill the sterilized bottles previously evacuated from Millipore using air from the diving regulator. In addition, 5–10 g of MS consisting of soil, rocks, and sand was collected in sterile polycarbonate tubes at a distance of 15 cm from the emission point using a sterilized plastic scoop. For these last samples, it was not possible to totally avoid seawater contamination. The HW, SW, and marine sediment samples were then stored at −20 °C.

### 2.2. Chemical–Physical Parameters

The temperature of the sampled HW was directly measured at the emission point, while pH, Eh, and electric conductivity were measured on the ship using portable instruments. In order to avoid any precipitation and dissolution of suspended particulate matter, all water samples were first filtered through cellulose filters (0.45 μm) and then acidified (about 90–100 microliters of 67–69% ultrapure HNO_3_ for trace analysis) for the determination of cations (Na^+^, K^+^, Ca^2+^, Mg^2+^) and minor and trace elements. Untreated samples were used for anion determination (Cl^−^, F^−^, Br^−^, NO^3−^, SO_4_^2−^). A chemical analysis of major constituents was carried out by ion chromatography (Dionex Dx 120; reproducibility ±2%; Thermo Fischer Scientific, Waltham, MA, USA), a Dionex AS14A (Thermo Fischer Scientific, Waltham, MA, USA) column was used for anions with ASRS (Anion Self-Regenerating Suppressor), and Dionex CS12A (Thermo Fischer Scientific, Waltham, MA, USA) was used for cations with CSRS (Cation Self-Regenerating Suppressor). HCO_3_ concentrations were determined in the laboratory by volumetric titration with HCl 0.1 N, with care taken to keep water samples in filled and sealed bottles to avoid their degassing.

Minor and trace elements were analyzed by ICP-MS (Agilent 7800ce; Headquarters 5301 Stevens Creek Blvd, Santa Clara, CA, USA); measurements were performed on 100 diluted filter-acidified water aliquots, spiked with an In-Re-Rh internal standard solution insert online just before they were nebulized for a total volume of 10 ppb. Isobaric and molecular interferences were minimized by a collision cell with He as an inert gas forming a collision gas interacting with the ion beam at higher pressures.

The accuracy of the determinations (±15%) was checked by analyzing certified multi-elemental reference water samples SPS-SW1 (LGC Queens Road Teddington, Middlesex, UK) and SPS-SW2 (LGC Queens Road Teddington, Middlesex, UK), with SRLS-4 (National Research Council of Canada) analyzed for the quality control of the instrument, both at the beginning and at the end of each analytical sequence.

### 2.3. Genomic DNA Extraction, PCR Amplification, and Sequencing

For metabarcoding analysis, 100 mL of the sample (triplicate) was filtered through cellulose filters (0.22 μm), and DNA was extracted from the filters using the DNeasy PowerWater Kit^®^ (QIAGEN, Strasse 1, Hilden, Germany) following the protocol suggested by the producer. For MS, 1 g of the sample (triplicate) was used to extract total metagenomic DNA with the PowerSoil^®^ DNA Isolation Kit (QIAGEN, Strasse 1, 40724 Hilden, Germany). Metagenomic DNA was verified by electrophoresis on 1% *w*/*v* agarose gel and quantified by a NanoDrop 2000c spectrophotometer (Thermo Fisher Scientific, Waltham, MA, USA). The extracted DNA was used to amplify the V3-V4 region of the 16S rRNA with universal primers (Pro341F: 5′-TCGTCGGCAGCGTCAGATGTGTATAAGAGACAGCCTACGGGNBGCASCAG-3′ and Pro805R: 5′-GTCTCGTGGGCTCGGAGATGTGTATAAGAGACAGGACTACNVGGGTATCTAATCC-3′) previously described [39] following the two-step PCR amplification protocol described in https://support.illumina.com/documents/documentation/chemistry_documentation/16s/16s-metagenomic-library-prep-guide-15044223-b.pdf (accessed on 5 May 2024). Libraries were sequenced in a 300 bp paired-end run on the Illumina MiSeq platform at IGA Technology Services s.r.l. (Udine, Italy).

### 2.4. Bioinformatic Analysis

An internal pipeline was created to analyze metabarcoding sequences. Read pairs were overlapped with flash v. 1.2.11 [40] with the parameter “--min-overlap 15” to generate consensus pseudo-reads, while non-overlapping reads were maintained as separated pairs. Both overlapping and non-overlapping reads were retained. Primer sequences used in amplification were removed with cutadapt v. 2.7 [41] with the following parameters: “discard-untrimmed minimum length 200 overlap 10 times 2 error-rate 0.15”. Low-quality bases at the 3’tails of reads were trimmed with erne-filter v. 1.4.3 [42] with the following parameters: “min-size 200”. QIIME pipeline v. 1.9.1 [43] was then executed. The library was scanned for the presence of chimeras with VSEARCH algorithm v. 2.14.1 [44]. The operational taxonomic unit (OTU) picking process was performed in “open-reference” mode against the SILVA v.138 SSU Ref NR 99 [45]. Taxonomy was assigned to OTUs using the RDP classifier v. 2.2 [46]. Only OTUs matching with a 97% minimum identity threshold and with a minimum confidence threshold of 0.50 were retained and subjected to further classification. The expression-based heatmap of correlation was generated using Spearman’s correlation test through the Heatmapper web server (http://www.heatmapper.ca/expression/, accessed on 15 March 2024).

### 2.5. Statistical Analysis

PERMANOVA (permutational analysis of variance) was performed with the null hypothesis stating that there is no significant difference among different types of samples (“type”, a fixed and orthogonal factor with three levels: hydrothermal water, seawater, and marine sediment) and different sampling dates (“date”, a fixed and orthogonal factor with two levels: May and August), among all the detected samples. A *p*-value analysis was applied to estimate the variability between the six samples at the phylum, class, and order levels. Data were considered significant for *p*-values < 0.05. Principal Coordinate Analysis (PCoA) was performed to evaluate the variations among samples using the Bray–Curtis distance matrix. Alpha diversity metrics, including the abundance-based coverage estimator (ACE), Chao1, Shannon–Wiener diversity index (H’), Simpson index (1-D), and evenness (e), were calculated to determine the specific microbial richness and diversity. Good’s coverage was estimated to assess the comprehensiveness of sampling.

## 3. Results

### 3.1. Chemical–Physical Characterization of the Samples

**Figure 2:**
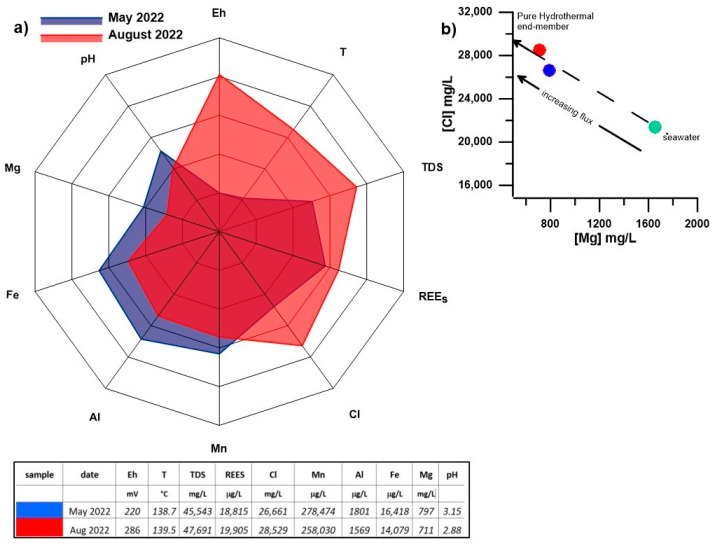
(**a**) Radar plot of main chemical–physical parameters and major and trace elements of May and August hydrothermal fluid samples; (**b**) [Mg^2+^] versus [Cl^−^] binary plot of hydrothermal samples (red and blue dots) compared with seawater (green dot).

The hot fluid samples collected in May and August, following the method described in Section 2.1 in order to avoid any water contamination, showed notable differences in their physical and chemical composition, especially in Total Dissolved Solids (TDS, expressed in g/L) values and rare earth element (REE) enrichment (see Table 1). The August samples were characterized by higher temperature and Eh values, lower pH (Figure 2; see Table 1), and a marked increase in Ca^2+^ and Cl^−^ concentration, accompanied by a decrease in Mg^2+^, Al^3+^, Mn^2+^, and Fe^2+^ compared to the May samples. In August, seawater samples collected on the seafloor, approximately 5 m from the hot spring, revealed striking anomalies in chemical–physical parameters, including an acid pH significantly lower than that of seawater. Additionally, despite the observed variation, the seawater temperature at this depth was notably high, indicating a significant influence of the hydrothermal spring that is purged from the seafloor to the surrounding marine environment.

### 3.2. Composition of Bacterial Communities Residing in BP in Different Seasons

Excluding the unclassified families, a total of 60 phyla, 122 classes, 242 orders, and 386 families were detected in different samples. The OTUs were successfully identified and classified at the species level using a 97% sequence similarity threshold against the “SILVA” database (Supplementary Table S1).

Rarefaction curves show a good level of diversity sampling, as confirmed by the Good’s coverage index for all the samples (Table 2 and Supplementary Figure S1). Furthermore, the Shannon–Wiener diversity index was, on average, 2.79 ± 0.6. The Simpson index ranged between 0.04 and 0.35, while evenness ranged between 0.34 and 0.72 (Table 2).

**Figure 3:**
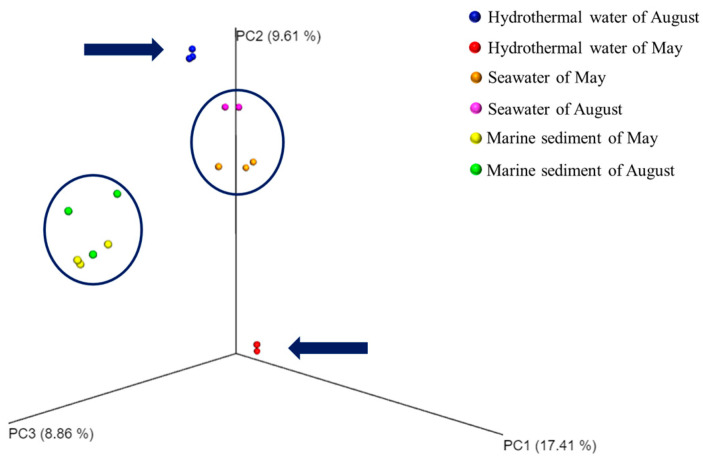
Principal Coordinate Analysis (PCoA) plot of all the samples analyzed using Bray–Curtis similarity. The arrows indicate the HW samples.

The PCoA plot indicates that the three replicas of each sample were very similar (Figure 3). Surprisingly, a shared microbial community, independent of the sampling expedition, was found in the SW and MS samples. In contrast, PCoA analysis revealed a significant difference between the two HW samples collected in May and August.

### 3.3. Taxonomic Composition of Prokaryotic Communities of BP Phyla

A comparison of the prokaryotic communities of BP phyla between the two sampling expeditions revealed that August samples exhibited greater bacterial biodiversity compared to those collected in May.

**Figure 4:**
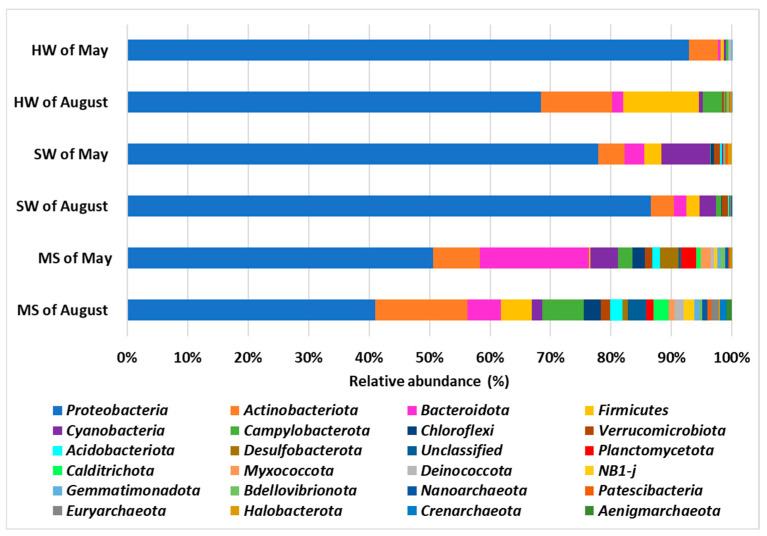
Percentage of the relative abundance averages of the 25 main phyla found in the samples of this study: hydrothermal water (HW); seawater (SW); and marine sediment (MS).

Proteobacteria were the predominant phylum in the microbial community of all samples (Figure 4 and Figure S2). They were abundant in both of the HW samples, with relative abundances of 93% and 68% for May and August, respectively, and in both the SW samples, with relative abundances of 78% and 86%, respectively. These percentages are lower (50% and 40%) in both MS samples collected in May and August, respectively.

Actinobacteriota were the second-most represented phylum in HW collected in August (12%) compared to May (5%) and in MS collected in August, with a relative abundance of 15%, while they were less abundant in MS collected in May (8%) and in both SW samples (4.4% and 4%, respectively, for May and August).

Bacteroidota were found to be more abundant in the MS sample collected in May (18%) than in all the other samples, where the relative percentages range from 0.4 to 5.5%.

The Firmicutes and the Campylobacterota phyla were more abundant in two out of the three samples collected in August, with relative abundances of 12% and 3.1% in HW and 4.9% and 6.7% in MS, suggesting the influence of bacteria present in the HW on the surrounding environment, since these phyla were poorly represented in both of the SW samples (0.3–2.9%).

Cyanobacteria were mainly present in both SW (8%) and MS collected in May (4.4%). These findings underscore the variability in microbial community composition across different sample types and sampling periods.

However, the HW of August was weirdly enriched with Firmicutes and Campylobacterota. In SW samples, Cyanobacteria were also represented. Additionally, shared phyla including Proteobacteria, Actinobacteria, Chloroflexi, and Bacteroidota were identified in MS; some phyla appeared to be mainly present in the MS of May, such as Cyanobacteria and Desulfobacterota, while others, such as Firmicutes and Campylobacterota, were mainly present in MS collected in August.

PERMANOVA was performed at the phyla level, considering the three types of samples (hydrothermal water, seawater, and marine sediment) and the two sampling expeditions (May and August). The analysis revealed a significant statistical difference in microbial composition based on both the sample type and the sampling expedition (*p* < 0.05) (Table 3).

### 3.4. Taxonomic Composition of Prokaryotic Communities of BP Classes and Orders

**Figure 5:**
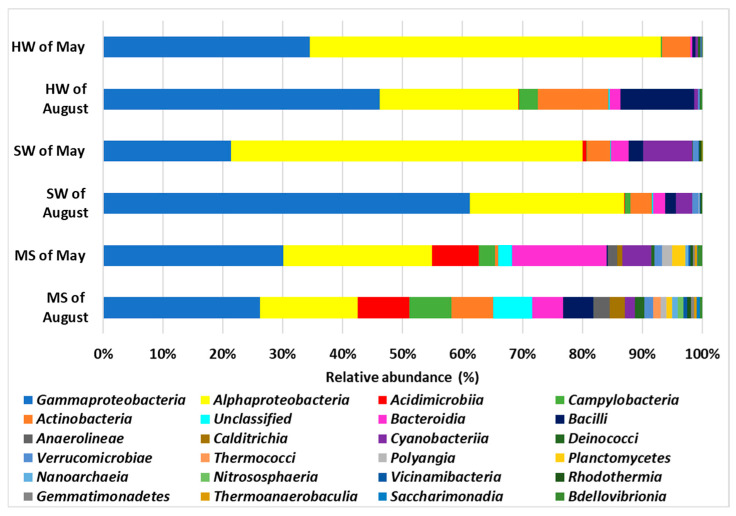
Percentage of relative abundance averages of the 25 main classes found in all samples of this study: hydrothermal water (HW); seawater (SW); marine sediment (MS).

At the class level, Alphaproteobacteria was the most abundant taxon in both HW and SW samples collected in May, with relative abundances of 58% and 57%. On the other hand, Gammaproteobacteria dominated the microbial community in both HW and SW samples collected in August, with relative abundances of 45% and 61%, respectively (Figure 5 and Figure S3). However, the ratio between the Alphaproteobacteria and Gammaproteobacteria classes was approximately the same in the MS samples between May and August (Figure 5 and Figure S3). Actinobacteria were more abundant in both the HW samples and the MS of August, with relative abundances of 12% and 7%, respectively. Additionally, they were present in both SW samples, with a relative percentage of 3.5–3.7%, and in the MS of May, with a relative percentage of 0.5%. The Bacteroidia class was more abundant in the MS of May, followed by in the MS of August, with relative abundances of 14% and 5%, respectively. However, this class was less abundant in all the other samples. The Bacilli class was mainly present in the August HW sample, with a relative abundance of 12%. Acidimicrobiia (7% and 8%) were highly abundant in both MS samples and less abundant in the other samples. Camplylobacteria were more present in the August HW and MS samples (2.5% and 6.7%). The Cyanobacteria class was more abundant in the May SW and MS samples, and it was less abundant in the May HW samples and in the other samples.

PERMANOVA was performed using the detected classes within the three types of samples (hydrothermal water, seawater, and marine sediment) and the two sampling expeditions (May and August). The analysis showed a significant statistical difference in microbial composition based on both the sample type and the sampling expedition (*p* < 0.001) (Table 4).

At the level of order, the MS samples shared the same bacteria, while more striking differences were found in HW and SW samples between May and August.

**Figure 6:**
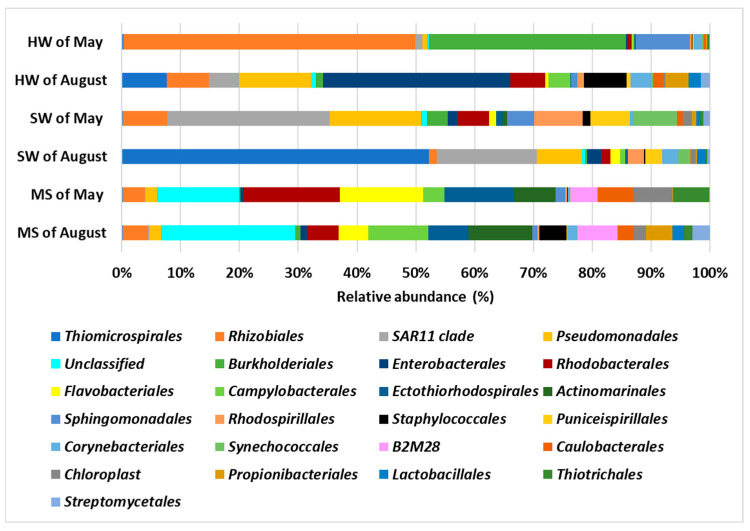
Percentage of relative abundance averages of the 25 main orders found in all samples of this study: hydrothermal water (HW); seawater (SW); marine sediment (MS).

Rhizobiales and Burkholderiales were predominant in HW in May, with relative abundances of 47% and 32% (Figure 6 and Figure S4), while Enterobacterales were predominant in HW in August, with a relative abundance of 27%.

The SW of May was mainly dominated by SAR11clade, with a relative abundance of 25%, and Pseudomonadales, with a relative abundance of 14%. The SW of August was highly abundant in Thiomicrospirales, with a relative abundance of 50%, followed by 16% and 7% for SAR11clade and Pseudomonadales. In the MS of May, various orders were found, such as Rhodobacterales, with a relative abundance of 11%, and Flavobacteriales (with a relative abundance of 9%), followed by Ectothiorhodospirales and Actinomarinales (with relative abundances of 8% and 5%, respectively). Other orders were less represented. The most represented orders in the MS of August, excluding the unclassified, were Actinomarinales and Campylobacterales, both with a relative abundance of 7%. Other orders were less represented.

PERMANOVA was used to detect significant differences at the order level among the three types of samples (hydrothermal water, seawater, and marine sediment) and across the two sampling expeditions (May and August). The analysis indicated a significant statistical difference in microbial composition related to both the sample type and the sampling expedition (*p* < 0.05) (Table 5).

### 3.5. Taxonomic Composition of Prokaryotic Communities of BP Families and Genera

**Figure 7:**
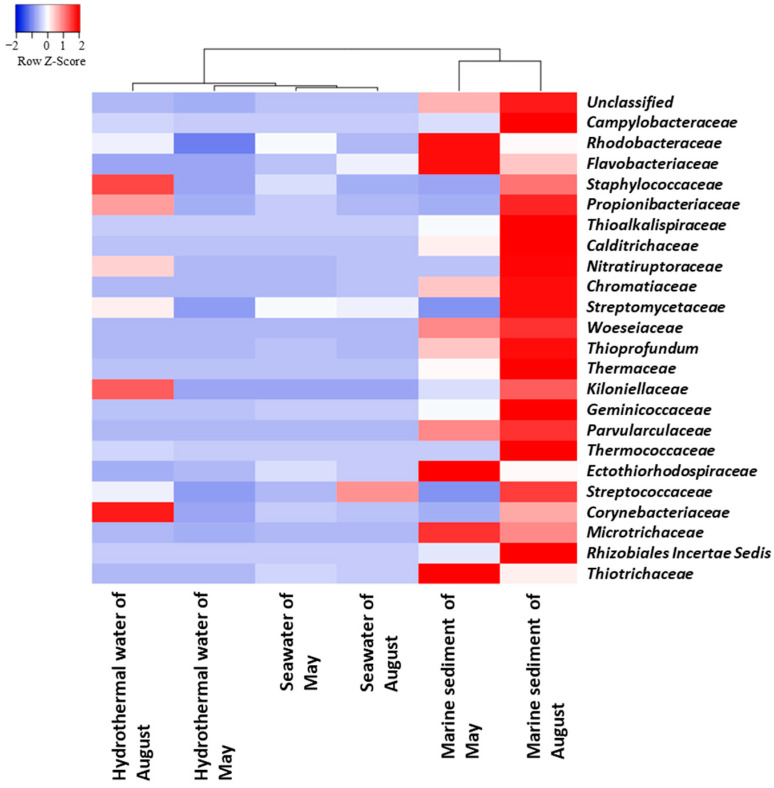
Heatmap based on the 25 main families detected in all the studied samples, generated by “Average Linkage” calculation using Spearman’s rank correlation.

At the family level, a heatmap analysis was performed to gain further insights into the microbial community composition across the different types of samples and sampling expeditions. This analysis reveals two groups based on their microbial community composition (Figure 7 and Figure S5): one contains the SW and HW samples from both sampling expeditions (May and August) and the other includes both of the MS samples and forms an exclusive group. SW samples were confirmed to be very similar and create a distinct subgroup within the larger group. However, the two HW samples are different from each other and form two distinct subgroups. The results highlight the diversity of microbial communities in HW compared to other sample types. The differences observed between hydrothermal water (HW) samples collected during various sampling expeditions highlight the dynamic nature of microbial communities as they respond to changes in hydrothermal fluids. The heatmap analysis supports the findings of the PCoA plot, underscoring the diversity of microbial communities in HW samples compared to other samples.

**Figure 8:**
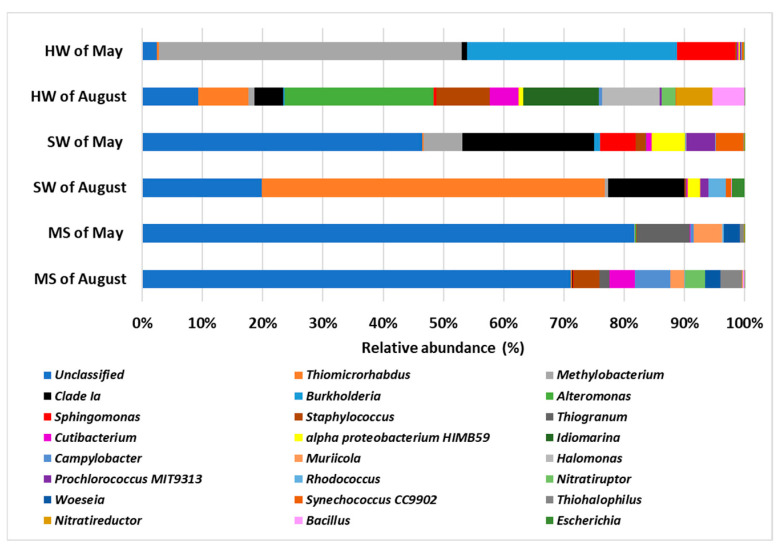
Percentage of relative abundance averages of the 25 main genera found in all samples of this study: hydrothermal water (HW); seawater (SW); marine sediment (MS).

Significant differences were observed in the microbial community composition at the genus level across all samples (Figure 8 and Figure S6). Although unclassified genera were highly abundant, certain distinctive genera were identified in specific samples. For instance, in the HW samples collected in May, *Methylobacterium* was the most abundant genus, followed by *Burkholderia*, with relative abundances of 44.36% and 30.76%, respectively. In contrast, the HW samples from August were dominated by *Alteromonas*, followed by *Idiomarina* and *Halomonas*, with relative abundances of 16.87%, 8.57%, and 6.51%, respectively.

In the SW samples collected in May, excluding the unclassified genera, the most abundant genera were *Clade* Ia and *Methylobacterium*, with relative abundances of 32.7% and 4.5%, respectively. However, in the SW samples from August, *Thiomicrorhabdus* emerged as the most abundant genus, followed by *Clade* Ia, with relative abundances of 49.6% and 10.9%, respectively.

Finally, unclassified genera dominated the microbial community in the MS samples from both months. Nonetheless, distinctive genera were identified: *Thiogranum* was prominent in the May MS samples (5.72%), while *Campylobacter* was notable in the August MS samples (4%).

## 4. Discussion

This study, for the first time, investigated the variation in the microbial community at Black Point across two sampling expeditions, in a unique hydrothermal vent near Panarea Island (Messina, Italy). Hydrothermal ecosystems are extreme habitats characterized by high and fluctuating temperatures and metal concentrations, which vary based on site-specific conditions. Key environmental factors, such as temperature, organic matter concentration, metal composition, and redox potential, drive community dynamics in these ecosystems [33]. These environments are biodiversity hotspots, where bacteria play a crucial role in maintaining ecosystem homeostasis. We analyzed the variations in microbial communities and physicochemical parameters at Black Point during two sampling campaigns (May and August). While previous studies have focused on the microbial communities of shallow-water hydrothermal vents in this region [31,32,34,35,36,37], our research provides the first characterization of the bacterial composition of hydrothermal water collected directly from the vent.

Although we ensured careful sample collection following the procedure described in [47], some limitations should be considered. These include the potential effects of sample storage at −20 °C prior to laboratory analysis, the choice of primer pairs for the amplification of the 16S rRNA gene, and the data analysis pipeline. Additionally, fluid mixing between vent emissions, surrounding seawater, and marine sediment cannot be ruled out.

Despite these limitations, our study provides a foundational milestone for future comparative analyses and offers initial insights into the chemical–physical and microbiological characteristics of Black Point.

In August, the samples collected from the hydrothermal vent and the nearby seafloor reveal significant differences in chemical–physical properties, indicating an overall increase in hot fluid output (Table 1, Figure 2).

This increased flow, as a primary result, leads to the enhancement of the pure hydrothermal end-member, equilibrated at a depth of 23 m [48,49,50], with its high salinity and typical hot (140 °C) and acidic (pH 2.0) fingerprint, that is less contaminated by seawater. The high Cl concentration is evidence of enrichment due to water–rock interaction enhanced by sweltering hot and acidic conditions. At the same time, a lower concentration of Mg, in the context of the May sample, is in good agreement with a more mature hydrothermal component. Furthermore, a higher concentration of REEs accompanies the low pH observed in August. Several processes control REEs in hydrothermal waters, including water–rock interactions and the composition of both rock and water, influencing the complexation and fractionation of REEs.

Additionally, an adsorption process, dependent on pH, occurs on the newly formed phases of Fe^2+^, Al^3+^, and Mn^2+^ oxy-hydroxides. Independently from the temperature and type of local rocks, REE concentrations in hydrothermal fluids are usually inversely correlated with pH [51], and scavenging by Fe^2+^ and Mn^2+^ oxy-hydroxides occurs at the vent–seafloor interface due to mixing with seawater [27,52,53].

In BP hydrothermal water, REE behavior is controlled by Fe, Al, and Mn oxy-hydroxides because this solid phase’s dissolution and precipitation process, including the sorption and desorption of REEs, is pH-dependent.

The positive correlations between the total amount of REEs and Fe^2+^, Al^3+^, and Mn^2+^ dissolved in water highlight the simultaneous variation in these elements, indicating the involvement of Fe, Al, and Mn in controlling the abundance of REE dissolved in water. On the contrary, pH values negatively correlate with Fe^2+^, Al^3+^, and Mn^2+^, indicating that dissolution and precipitation processes control rare earth element (REE) behavior. As a result, a vigorous scavenging process occurs during co-precipitation and adsorption onto the surfaces of Fe^2+^, Al^3+^, and Mn^2+^ oxides and oxy-hydroxides [52,54,55].

The radar plot in Figure 2 well describes the divergent features of the two hot fluid samples. Indeed, in August, a general increase in pressure in the hydrothermal field, likely generated by a deep input from the feeding system, resulted in general output variation in the gas vents and the hot fluids being purged from BP and the surrounding area. The physical condition of the seawater sample collected at a depth of 23 m far from BP testifies to an aerial extension of the hot fluid flux that, flowing out from seafloor sediment, heated and contaminated the seawater column above. The binary plot of magnesium versus chloride in Figure 2b represents the chemical properties of a pure hydrothermal component. It shows that as the flux output increases, there is progressively less contamination by seawater, approaching the ideal condition of zero magnesium in the pure end-member.

At the same time, our results suggest significant changes in the microbial community between the May and August samples of hydrothermal water. In contrast, the microbial community of seawater and marine sediment shows greater resilience, exhibiting less pronounced changes compared to the other samples. Similar results were found in another study [56].

A previous study [33] reported on the prokaryotic community at BP using Illumina Sequencing Technology, with some technical differences compared to our approach, such as the type of water sampled, the primer pairs employed, and the bioinformatics pipeline used to analyze bacterial composition.

Despite these differences, comparable results were obtained. Similarly to the findings of the mentioned study and earlier reports [31,32,33,57], the bacterial composition was dominated by bacteria over archaea, even in the high-temperature conditions where sampling was conducted.

The dominance of Proteobacteria, variations in Actinobacteriota abundance, and the differences in Bacteroidota, Firmicutes, and Campylobacterota highlight the complex dynamics of microbial communities in BP ecosystems as previously reported [33]. Indeed, other studies found high variability within the Proteobacteria phylum [58] and underlined the different compositions of microbial communities around the world [59].

In this study, we observed a shift within Proteobacteria, particularly in the Gammaproteobacteria and Alphaproteobacteria classes, between the May and August samples, which highlights the dynamic nature of microbial communities in these peculiar marine environments and the influence of environmental factors on their composition [7,60].

Our results align with previous studies that emphasized the significant roles of Gammaproteobacteria in hydrogen and sulfur oxidation (SUP05 group) and methane oxidation (Methylothermaceae) in hydrothermal water. However, in other studies, Epsilonproteobacteria were more abundant in hydrothermal water [9,33,61,62]. The lower abundance of these particular classes in our study could be related to BP’s lower sulfide levels than other hydrothermal waters, which typically contain higher sulfide concentrations. This correlation between class distribution and sulfide levels might explain why these classes are less prevalent in our samples.

In our study, Gammaproteobacteria and Campylobacterota were the most abundant classes in the hydrothermal water of August, consistent with previous studies highlighting the prevalence of Campylobacterota in acidic and turbulent environments [63]. Furthermore, another study suggested that Campylobacterota inhabited shallow marine waters during the early stages, followed by Gammaproteobacteria [64], following chemical–physical variation in the fluids. This differential distribution trend may be associated with variations in sulfide and oxygen levels and temporal factors [64]. In our study, the exceptional sampling period, especially in August, in which hydrothermal water leakage was more abundant, explains the increased abundance of these classes. Moreover, in our study, Campylobacterales, especially the *Sulfurimonas* and *Sulfurovum* genera, were more abundant in hydrothermal water in August. These genera are known for carbon fixation under moderate and extreme acidic conditions and may significantly contribute to overall hydrogen consumption in deep-sea vent systems [63,65,66,67].

Further investigation is necessary to elucidate the differential prevalence of Rhizobiales, Sphingomonadales, and Burkholderiales in hydrothermal water and seawater in May.

All samples collected in August showed more diversity in their microbial communities compared to those collected in May. These findings challenge studies in which temperature within the chimney is a critical factor influencing microbial colonization and vents with high-temperature sites (>80 °C) have lower diversity compared to lower- and moderate-temperature sites [37,68]. Furthermore, pH has been identified as a significant influencer of microbial composition, as Arcadi et al. 2023 [36] reported. Our study supports this finding, revealing a decreased abundance of Proteobacteria and Bacteroidota in the samples collected in August, where the pH was more acidic, alongside an increased prevalence of Campylobacterota.

In August hydrothermal water samples, the most abundant orders were Enterobacterales, Pseudomonadales, and Thiomicospirales. Our results are in accordance with previous studies, in which Enterobacterales were found in marine sediments, waters, and shallow-water sponges as epibionts, and underline the presence of these bacteria in the deep sea, where they efficiently reduce Fe (III). Even though these organisms are primarily associated with anoxic environments, where they either reduce sulfate or rely on fermentation, they could also play a role in recycling organic matter in oxygen-rich settings [69].

Pseudomonadales have been isolated from marine sediment associated with two deep-sea hydrothermal vents and are reported in other studies in the degradation of toluene and benzene [63,70]. Furthermore, other studies have frequently reported the presence of the Thiomicospirales order in hydrothermal chimneys and sediments, where acidic fluids containing abundant carbon dioxide (CO_2_) correlate positively with nitrate [71,72]. Indeed, in our study, this order was more abundant in August than in May, suggesting that the presence of this order is correlated with the more acidic pH and the greater abundance of NO_3_.

Additionally, the abundance of specific orders, such as Rhizobiales, SAR11clade, and Thiomicrospirales in some samples, underscores the importance of understanding the ecological roles of these microbial taxa in their respective environments [73]. SAR11, Puniceispirillales (SAR116), and Rhodobacterales are widespread across oceans. SAR11 is a group of carbon-oxidizing bacteria characterized by their aerobic, free-living, and chemoheterotrophic nature and are particularly abundant [74]. Other studies have reported that Rhodospirillales, such as anaerobic photosynthetic bacteria, engage in nitrogen and sulfur metabolism, such as sulfur oxidation, cysteine synthesis, and glutamine/glutamate synthesis. Additionally, researchers have recovered numerous novel isolates belonging to this class from the deep sea and associated them with petroleum hydrocarbon degradation [75,76]. Moreover, Rhodobacterales have been known for their roles in nitrogen and sulfur metabolism, including sulfur oxidation, cysteine synthesis, and glutamine/glutamate synthesis [77].

The most common Bacteroidota genera include Flavobacteriales, primarily aerobic or facultative aerobic bacteria with strict chem-organic heterotrophic metabolism [78]. On the other hand, members of *Ectothiorhodospiraceae* display a wide range of metabolic capabilities, engaging in photolithotrophy, photoheterotrophy, chemoheterotrophy, chemolithotrophy, and methylotrophy [79]. They use different electron acceptors, including nitrite and sulfur compounds, and thrive in iron-rich environments. These orders appeared in greater abundance in the marine sediment collected in May, indicating that a high concentration of chemical and physical elements, such as iron, might affect the microbial composition of entire ecosystems.

Meanwhile, Actinomarinales was the most prevalent order in the microbial community of marine sediment gathered in August. Members of this order oxidize organic matter into CO_2_ through the tricarboxylic acid (TCA) cycle, and they are known to live in marine, hydrothermal, and freshwater sediments. These bacteria also carry genes that encode transporters for ferric ions, ammonium, phosphate, and phosphonates [80,81].

The genus *Methylobacterium*, which was highly abundant in the HW samples collected in May in this study, has also been identified in various natural environments, including coastal and pelagic seawater, as well as deep-sea hydrothermal vents. As noted by Kato et al. (2009) [82], this genus utilizes compounds such as formate and methanol for growth. In contrast, the dominant genus in the HW samples from August was *Alteromonas*, which is known for producing polyhydroxyalkanoates (PHAs) and exopolysaccharides (EPSs). It also plays a significant role in the degradation of organic matter in marine environments.

In the SW samples collected in August, the most abundant genus was *Thiomicrorhabdus*, which has been reported in various environments, including deep-sea hydrothermal vents. Recently, new species of *Thiomicrorhabdus*, such as *Thiomicrorhabdus indica*, have been isolated from hydrothermal vents. These species can grow using thiosulfate, sulfide, elemental sulfur, or tetrathionate as their sole energy source [83].

## 5. Conclusions

This study provides novel insights into the microbial communities within the hydrothermal vent ecosystem of Black Point near Panarea Island, Italy. By examining samples collected in May and August, we observed significant variations in microbial abundance and composition across hydrothermal water, seawater, and marine sediment.

The dominance of specific microbial taxa varied between sampling expeditions, highlighting the influence of temporal factors and environmental parameters, such as temperature, pH, and chemical composition.

Overall, our findings can contribute to the understanding of the ecological role of microbial communities in hydrothermal vent ecosystems and underscore the importance of considering temporal dynamics and environmental parameters in microbial ecology research. Further investigations are needed to elucidate the specific metabolic activities and interactions driving microbial community dynamics in these extreme environments.

## Figures and Tables

**Table 1:** Chemical–physical parameters of the samples analyzed in this study.

	Hydrothermal Water (HW)		Seawater (SW) 3	
	pH	Temperature °C	pH	Temperature °C
May	3.15	138.5	7.96	15
August	2.88	139.5	6.78	27

**Table 2:** Descriptions and diversity indices of the samples used in this study.

Sample	S	Good’s Coverage	Chao1	α	1-D	H’	e
HW of May	160	0.97	1089.764	183.34	0.29	1.84	0.36
	128	0.97	1045.358	161.95	0.30	1.78	0.37
	110	0.97	849.734	154.65	0.35	1.58	0.34
HW of August	118	0.96	1303.493	167.34	0.07	3.30	0.69
	112	0.97	1095.135	190.66	0.08	3.00	0.67
	109	0.96	1488.415	245.11	0.06	3.36	0.72
SW of May	120	0.96	1163.985	231.73	0.08	2.97	0.62
	174	0.95	1497.837	167.48	0.08	2.95	0.57
	155	0.95	1675.768	129.65	0.04	3.58	0.71
SW of August	138	0.96	1473.207	257.10	0.10	3.11	0.63
	201	0.96	1356.164	310.61	0.35	1.98	0.37
	186	0.96	1313.642	269.91	0.34	2.00	0.38
MS of May	165	0.95	1893.452	123.24	0.09	3.19	0.63
	203	0.94	2113.745	180.50	0.10	3.13	0.59
	192	0.94	1969.215	169.60	0.10	3.25	0.62
MS of August	185	0.93	2372.832	202.52	0.16	2.96	0.57
	135	0.97	1053.659	257.03	0.12	3.02	0.62
	213	0.92	2488.969	214.95	0.12	3.31	0.62

S represents the total number of bacterial families; Chao1 is an abundance-based richness estimator; α is the alpha diversity; 1-D is the Simpson’s index; H’ is the Shannon–Wiener diversity; e is the evenness.

**Table 3:** PERMANOVA of microbial community found at phyla level.

Source	DF	Adj. SS	Adj. MS	F-Value	*p*-Value
Samples	2	7078.4	3539.2	10.995	0.001
Sampling expeditions	3	2574.8	858.26	2.6663	0.007
Res	12	3862.7	321.89		
Total	17	13,516			

DF—total degrees of freedom; Adj. SS—adjusted sums of squares; Adj. MS—adjusted mean squares. *p*-value is significant (*p* < 0.05).

**Table 4:** PERMANOVA of microbial community found at class level.

Source	DF	Adj. SS	Adj. MS	F-Value	*p*-Value
Samples	2	8331.9	4165.9	11.601	0.001
Sampling expeditions	3	3572.8	1190.9	3.3163	0.001
Res	12	4309.4	359.11		
Total	17	16,214			

DF—total degrees of freedom; Adj. SS—adjusted sums of squares; Adj. MS—adjusted mean squares. *p*-value is significant (*p* < 0.05).

**Table 5:** PERMANOVA of microbial community found at order level.

Source	DF	Adj. SS	Adj. MS	F-Value	*p*-Value
Samples	2	14456	7228.2	13.91	0.001
Sampling expeditions	3	7623.4	2541.1	4.8902	0.001
Res	12	6235.6	519.64		
Total	17	28,315			

DF—total degrees of freedom; Adj. SS—adjusted sums of squares; Adj. MS—adjusted mean squares. *p*-value is significant (*p* < 0.05).

## Data Availability

The datasets analyzed during the current study are available in the GenBank database with the following accession number and BioProject ID: PRJNA1190326.

## Supplementary Materials

The following supporting information can be downloaded at https://www.mdpi.com/article/10.3390/biology14010086/s1, Figure S1. Rarefaction curves of the samples analyzed in this study. Table S1. Total number of operational taxonomic units (OTUs) resulting from the pipeline dataset. Figure S2. Percentage of relative abundance of the 25 main phyla found in all samples of this study. Figure S3. Percentage of relative abundance of the 25 main classes found in all samples of this study. Figure S4. Percentage of relative abundance of the 25 main orders found in all samples of this study. Figure S5. Percentage of relative abundance of the 25 main families found in all samples of this study. Figure S6. Percentage of relative abundance of the 25 main genera found in all samples of this study.

## References

[1] Bagnato E.B., Oliveri E., Acquavita A., Covelli S., Petranich E., Barra M., Italiano F., Parello F., Sprovieri M. (2017). Hydrochemical Mercury Distribution and Air-Sea Exchange over the Submarine Hydrothermal Vents off-Shore Panarea Island (Aeolian Arc, Tyrrhenian Sea). Mar. Chem..

[2] Resing J.A., Baker E.T., Lupton J.E., Walker S.L., Butterfield D.A., Massoth G.J., Nakamura K. (2009). Chemistry of Hydrothermal Plumes above Submarine Volcanoes of the Mariana Arc. Geochem. Geophys. Geosystems.

[3] Solomon E.A., Kastner M., Wheat C.G., Jannasch H., Robertson G., Davis E.E., Morris J.D. (2009). Long-Term Hydrogeochemical Records in the Oceanic Basement and Forearc Prism at the Costa Rica Subduction Zone. Earth Planet. Sci. Lett..

[4] Voordeckers J.W., Do M.H., Hügler M., Ko V., Sievert S.M., Vetriani C. (2008). Culture Dependent and Independent Analyses of 16S rRNA and ATP Citrate Lyase Genes: A Comparison of Microbial Communities from Different Black Smoker Chimneys on the Mid-Atlantic Ridge. Extremophiles.

[5] Van Der Most N., Qian P.-Y., Gao Y., Gollner S. (2023). Active Hydrothermal Vent Ecosystems in the Indian Ocean Are in Need of Protection. Front. Mar. Sci..

[6] Zhang L., Kang M., Xu J., Xu J., Shuai Y., Zhou X., Yang Z., Ma K. (2016). Bacterial and Archaeal Communities in the Deep-Sea Sediments of Inactive Hydrothermal Vents in the Southwest India Ridge. Sci. Rep..

[7] He A., Yu T. (2022). Changes in Microbial Communities at Deep-Sea Hydrothermal Vents during Active and Inactive Periods. Highlights Sci. Eng. Technol..

[8] Dick G.J. (2019). The Microbiomes of Deep-Sea Hydrothermal Vents: Distributed Globally, Shaped Locally. Nat. Rev. Microbiol..

[9] Rizzo C., Arcadi E., Calogero R., Sciutteri V., Consoli P., Esposito V., Canese S., Andaloro F., Romeo T. (2022). Ecological and Biotechnological Relevance of Mediterranean Hydrothermal Vent Systems. Minerals.

[10] Li J., Cheng H., Yin F., Liu J., Zhang X.-H., Yu M. (2024). Deciphering Microbial Communities and Distinct Metabolic Pathways in the Tangyin Hydrothermal Fields of Okinawa Trough through Metagenomic and Genomic Analyses. Microorganisms.

[11] Fullerton H., Smith L., Enriquez A., Butterfield D., Wheat C.G., Moyer C.L. (2024). Seafloor Incubation Experiments at Deep-Sea Hydrothermal Vents Reveal Distinct Biogeographic Signatures of Autotrophic Communities. FEMS Microbiol. Ecol..

[12] Beccaluva L., Gabbianelli G., Lucchini F., Rossi P.L., Savelli C. (1985). Petrology and K/Ar Ages of Volcanics Dredged from the Eolian Seamounts: Implications for Geodynamic Evolution of the Southern Tyrrhenian Basin. Earth Planet. Sci. Lett..

[13] Beccaluva L., Rossi P.L., Serri G. (1982). Neogene to Recent Volcanism of the Southern Tyrrhenian-Sicilian Area; Implications for the Geodynamic Evolution of the Calabrian Arc. Earth Evol. Sci..

[14] Boccaletti M. (1978). The Tyrrhenian Sea and Adjoining Areas. The Ocean Basins and Margins.

[15] Favalli M., Pareschi M.T., Neri A., Isola I. (2005). Forecasting Lava Flow Paths by a Stochastic Approach. Geophys. Res. Lett..

[16] Gabbianelli G., Romagnoli C., Rossi P.L., Calanchi N. (1993). Marine geology of the Panarea-Stromboli area (Aeolian Archipelago, Southeastern Tyrrhenian Sea). Acta Vulcanol..

[17] Gabbianelli G., Gillot P.Y., Lanzafame G., Romagnoli C., Rossi P.L. (1990). Tectonic and Volcanic Evolution of Panarea (Aeolian Islands, Italy). Mar. Geol..

[18] Italiano F., Nuccio P.M. (1991). Geochemical Investigations of Submarine Volcanic Exhalations to the East of Panarea, Aeolian Islands, Italy. J. Volcanol. Geotherm. Res..

[19] Gamberi F., Marani M., Savelli C. (1997). Tectonic, Volcanic and Hydrothermal Features of a Submarine Portion of the Aeolian Arc (Tyrrhenian Sea). Mar. Geol..

[20] Capaccioni B., Tassi F., Vaselli O., Tedesco D., Poreda R. (2007). Submarine Gas Burst at Panarea Island (Southern Italy) on 3 November 2002: A Magmatic versus Hydrothermal Episode. J. Geophys. Res. Solid Earth.

[21] Caliro S., Caracausi A., Chiodini G., Ditta M., Italiano F., Longo M., Minopoli C., Nuccio P.M., Paonita A., Rizzo A. (2004). Evidence of a Recent Input of Magmatic Gases into the Quiescent Volcanic Edifice of Panarea, Aeolian Islands, Italy. Geophys. Res. Lett..

[22] Caracausi A., Ditta M., Italiano F., Longo M., Nuccio P.M., Paonita A. (2005). Massive Submarine Gas Output during the Volcanic Unrest off Panarea Island (Aeolian Arc, Italy): Inferences for Explosive Conditions. Geochem. J..

[23] Caracausi A., Ditta M., Italiano F., Longo M., Nuccio P.M., Paonita A., Rizzo A. (2005). Changes in Fluid Geochemistry and Physico-Chemical Conditions of Geothermal Systems Caused by Magmatic Input: The Recent Abrupt Outgassing off the Island of Panarea (Aeolian Islands, Italy). Geochim. Cosmochim. Acta.

[24] Tassi F., Capaccioni B., Caramanna G., Cinti D., Montegrossi G., Pizzino L., Quattrocchi F., Vaselli O. (2009). Low-pH Waters Discharging from Submarine Vents at Panarea Island (Aeolian Islands, Southern Italy) after the 2002 Gas Blast: Origin of Hydrothermal Fluids and Implications for Volcanic Surveillance. Appl. Geochem..

[25] Esposito A., Giordano G., Anzidei M. (2006). The 2002–2003 Submarine Gas Eruption at Panarea Volcano (Aeolian Islands, Italy): Volcanology of the Seafloor and Implications for the Hazard Scenario. Mar. Geol..

[26] Longo M., Lazzaro G., Caruso C.G., Radulescu V., Radulescu R., Sciré Scappuzzo S.S., Birot D., Italiano F. (2021). Black Sea Methane Flares From the Seafloor: Tracking Outgassing by Using Passive Acoustics. Front. Earth Sci..

[27] Bau M., Dulski P. (1999). Comparing Yttrium and Rare Earths in Hydrothermal Fluids from the Mid-Atlantic Ridge: Implications for Y and REE Behaviour during near-Vent Mixing and for the YrHo Ratio of Proterozoic Seawater. Chemical Geology.

[28] Halbach P. (1986). Processes Controlling the Heavy Metal Distribution in Pacific Ferromanganese Nodules and Crusts. Geol. Rundsch..

[29] Price R.E., LaRowe D.E., Italiano F., Savov I., Pichler T., Amend J.P. (2015). Subsurface Hydrothermal Processes and the Bioenergetics of Chemolithoautotrophy at the Shallow-Sea Vents off Panarea Island (Italy). Chem. Geol..

[30] Pedersen R.B., Rapp H.T., Thorseth I.H., Lilley M.D., Barriga F.J.A.S., Baumberger T., Flesland K., Fonseca R., Früh-Green G.L., Jorgensen S.L. (2010). Discovery of a Black Smoker Vent Field and Vent Fauna at the Arctic Mid-Ocean Ridge. Nat. Commun..

[31] Maugeri T.L., Lentini V., Gugliandolo C., Cousin S., Stackebrandt E. (2010). Microbial Diversity at a Hot, Shallow-Sea Hydrothermal Vent in the Southern Tyrrhenian Sea (Italy). Geomicrobiol. J..

[32] Maugeri T.L., Gugliandolo C., Lentini V. (2013). Diversity of Prokaryotes at a Shallow Submarine Vent of Panarea Island (Italy) by High-Throughput Sequencing. Atti Accad. Perloritana Pericolanti Cl. Sci. Fis. Mat. Nat..

[33] Lentini V., Gugliandolo C., Bunk B., Overmann J., Maugeri T.L. (2014). Diversity of Prokaryotic Community at a Shallow Marine Hydrothermal Site Elucidated by Illumina Sequencing Technology. Curr. Microbiol..

[34] Antranikian G., Suleiman M., Schäfers C., Adams M.W.W., Bartolucci S., Blamey J.M., Birkeland N.-K., Bonch-Osmolovskaya E., Da Costa M.S., Cowan D. (2017). Diversity of Bacteria and Archaea from Two Shallow Marine Hydrothermal Vents from Vulcano Island. Extremophiles.

[35] Gugliandolo C., Maugeri T.L. (2019). Phylogenetic Diversity of Archaea in Shallow Hydrothermal Vents of Eolian Islands, Italy. Diversity.

[36] Arcadi E., Buschi E., Rastelli E., Tangherlini M., De Luca P., Esposito V., Calogero R., Andaloro F., Romeo T., Danovaro R. (2023). Novel Insights on the Bacterial and Archaeal Diversity of the Panarea Shallow-Water Hydrothermal Vent Field. Microorganisms.

[37] Barosa B., Ferrillo A., Selci M., Giardina M., Bastianoni A., Correggia M., Di Iorio L., Bernardi G., Cascone M., Capuozzo R. (2023). Mapping the Microbial Diversity Associated with Different Geochemical Regimes in the Shallow-Water Hydrothermal Vents of the Aeolian Archipelago, Italy. Front. Microbiol..

[38] Voltattorni N., Caramanna G., Cinti D., Galli G., Pizzino L., Quattrocchi F. (2006). Study of natural CO_2_ emissions in different italian geological scenarios. Advances in the Geological Storage of Carbon Dioxide.

[39] Chen H., Mozzicafreddo M., Pierella E., Carletti V., Piersanti A., Ali S.M., Ame S.M., Wang C., Miceli C. (2021). Dissection of the Gut Microbiota in Mothers and Children with Chronic Trichuris Trichiura Infection in Pemba Island, Tanzania. Parasit. Vectors.

[40] Magoč T., Salzberg S.L. (2011). FLASH: Fast Length Adjustment of Short Reads to Improve Genome Assemblies. Bioinformatics.

[41] Martin M. (2011). Cutadapt Removes Adapter Sequences from High-Throughput Sequencing Reads. EMBnet. J..

[42] Del Fabbro C., Scalabrin S., Morgante M., Giorgi F.M. (2013). An Extensive Evaluation of Read Trimming Effects on Illumina NGS Data Analysis. PLoS ONE.

[43] Caporaso J.G., Kuczynski J., Stombaugh J., Bittinger K., Bushman F.D., Costello E.K., Fierer N., Peña A.G., Goodrich J.K., Gordon J.I. (2010). QIIME Allows Analysis of High-Throughput Community Sequencing Data. Nat. Methods.

[44] Rognes T., Flouri T., Nichols B., Quince C., Mahé F. (2016). VSEARCH: A Versatile Open Source Tool for Metagenomics. PeerJ.

[45] Quast C., Pruesse E., Yilmaz P., Gerken J., Schweer T., Yarza P., Peplies J., Glöckner F.O. (2012). The SILVA Ribosomal RNA Gene Database Project: Improved Data Processing and Web-Based Tools. Nucleic Acids Res..

[46] Wang Q., Garrity G.M., Tiedje J.M., Cole J.R. (2007). Naïve Bayesian Classifier for Rapid Assignment of rRNA Sequences into the New Bacterial Taxonomy. Appl. Environ. Microbiol..

[47] Gugliandolo C., Italiano F., Maugeri T. (2009). The Submarine Hydrothermal System of Panarea (Southern Italy): Biogeochemical Processes at the Thermal Fluids—Sea Bottom Interface. Ann. Geophys..

[48] Bischoff J.L., Rosenbauer R.J. (1984). The Critical Point and Twophase Boundary of Seawater, 200–500°C. Earth Planet. Sci. Lett..

[49] Bischoff J.L. (1980). Geothermal System at 21°N, East Pacific Rise: Physical Limits on Geothermal Fluid and Role of Adiabatic Expansion. Science.

[50] Von Damm K.L., Edmond J.M., Grant B., Measures C.I., Walden B., Weiss R.F. (1985). Chemistry of Submarine Hydrothermal Solutions at 21°N, East Pacific Rise. Geochim. Cosmochim. Acta.

[51] Michard A. (1989). Rare Earth Element Systematics in Hydrothermal Fluids. Geochim. Cosmochim. Acta.

[52] Inguaggiato C., Censi P., Zuddas P., Londoño J.M., Chacón Z., Alzate D., Brusca L., D’Alessandro W. (2015). Geochemistry of REE, Zr and Hf in a Wide Range of pH and Water Composition: The Nevado Del Ruiz Volcano-Hydrothermal System (Colombia). Chem. Geol..

[53] Wood S.A. (2005). The Geochemistry of Rare Earth Elements and Yttrium in Geothermal Waters.

[54] Bau M., Koschinsky A. (2009). Oxidative Scavenging of Cerium on Hydrous Fe Oxide: Evidence from the Distribution of Rare Earth Elements and Yttrium between Fe Oxides and Mn Oxides in Hydrogenetic Ferromanganese Crusts. Geochem. J..

[55] Censi P., Zuddas P., Larocca D., Saiano F., Placenti F., Bonanno A. (2007). Recognition of Water Masses According to Geochemical Signatures in the Central Mediterranean Sea: Y/Ho Ratio and Rare Earth Element Behaviour. Chem. Ecol..

[56] Shade A., Gregory Caporaso J., Handelsman J., Knight R., Fierer N. (2013). A Meta-Analysis of Changes in Bacterial and Archaeal Communities with Time. ISME J..

[57] Maugeri T.L., Lentini V., Gugliandolo C., Italiano F., Cousin S., Stackebrandt E. (2009). Bacterial and Archaeal Populations at Two Shallow Hydrothermal Vents off Panarea Island (Eolian Islands, Italy). Extremophiles.

[58] Meier D.V., Bach W., Girguis P.R., Gruber-Vodicka H.R., Reeves E.P., Richter M., Vidoudez C., Amann R., Meyerdierks A. (2016). Heterotrophic *Proteobacteria* in the Vicinity of Diffuse Hydrothermal Venting. Environ. Microbiol..

[59] Caramanna G., Sievert S.M., Bühring S.I. (2021). Submarine Shallow-Water Fluid Emissions and Their Geomicrobiological Imprint: A Global Overview. Front. Mar. Sci..

[60] Sucato A., Vecchioni L., Savoca D., Presentato A., Arculeo M., Alduina R. (2021). A Comparative Analysis of Aquatic and Polyethylene-Associated Antibiotic-Resistant Microbiota in the Mediterranean Sea. Biology.

[61] Nakagawa S., Takai K., Inagaki F., Hirayama H., Nunoura T., Horikoshi K., Sako Y. (2005). Distribution, Phylogenetic Diversity and Physiological Characteristics of Epsilon-*Proteobacteria* in a Deep-sea Hydrothermal Field. Environ. Microbiol..

[62] Zhou Z., Liu Y., Pan J., Cron B.R., Toner B.M., Anantharaman K., Breier J.A., Dick G.J., Li M. (2020). Gammaproteobacteria Mediating Utilization of Methyl-, Sulfur- and Petroleum Organic Compounds in Deep Ocean Hydrothermal Plumes. ISME J..

[63] Zhou Z., St. John E., Anantharaman K., Reysenbach A.-L. (2022). Global Patterns of Diversity and Metabolism of Microbial Communities in Deep-Sea Hydrothermal Vent Deposits. Microbiome.

[64] Patwardhan S., Foustoukos D.I., Giovannelli D., Yücel M., Vetriani C. (2018). Ecological Succession of Sulfur-Oxidizing Epsilon- and Gammaproteobacteria During Colonization of a Shallow-Water Gas Vent. Front. Microbiol..

[65] Adam N., Perner M. (2018). Microbially Mediated Hydrogen Cycling in Deep-Sea Hydrothermal Vents. Front. Microbiol..

[66] Deng W., Zhao Z., Li Y., Cao R., Chen M., Tang K., Wang D., Fan W., Hu A., Chen G. (2023). Strategies of Chemolithoautotrophs Adapting to High Temperature and Extremely Acidic Conditions in a Shallow Hydrothermal Ecosystem. Microbiome.

[67] McNichol J., Dyksma S., Mußmann M., Seewald J.S., Sylva S.P., Sievert S.M. (2022). Genus-Specific Carbon Fixation Activity Measurements Reveal Distinct Responses to Oxygen among Hydrothermal Vent *Campylobacteria*. Appl. Environ. Microbiol..

[68] Tang K., Zhang Y., Lin D., Han Y., Chen C.-T.A., Wang D., Lin Y.-S., Sun J., Zheng Q., Jiao N. (2018). Cultivation-Independent and Cultivation-Dependent Analysis of Microbes in the Shallow-Sea Hydrothermal System Off Kueishantao Island, Taiwan: Unmasking Heterotrophic Bacterial Diversity and Functional Capacity. Front. Microbiol..

[69] Leinberger J., Milke F., Christodoulou M., Poehlein A., Caraveo-Patiño J., Teske A., Brinkhoff T. (2022). Microbial Epibiotic Community of the Deep-Sea Galatheid Squat Lobster Munidopsis Alvisca. Sci. Rep..

[70] Sun Q., Wang M., Sun L. (2015). Characteristics of the Cultivable Bacteria from Sediments Associated with Two Deep-Sea Hydrothermal Vents in Okinawa Trough. World J. Microbiol. Biotechnol..

[71] Brazelton W.J., Baross J.A. (2010). Metagenomic Comparison of Two Thiomicrospira Lineages Inhabiting Contrasting Deep-Sea Hydrothermal Environments. PLoS ONE.

[72] Muck S., De Corte D., Clifford E.L., Bayer B., Herndl G.J., Sintes E. (2019). Niche Differentiation of Aerobic and Anaerobic Ammonia Oxidizers in a High Latitude Deep Oxygen Minimum Zone. Front. Microbiol..

[73] Sheik C.S., Anantharaman K., Breier J.A., Sylvan J.B., Edwards K.J., Dick G.J. (2015). Spatially Resolved Sampling Reveals Dynamic Microbial Communities in Rising Hydrothermal Plumes across a Back-Arc Basin. ISME J..

[74] Giovannoni S.J. (2017). SAR11 Bacteria: The Most Abundant Plankton in the Oceans. Annu. Rev. Mar. Sci..

[75] Coelho F.J.R.C., Louvado A., Domingues P.M., Cleary D.F.R., Ferreira M., Almeida A., Cunha M.R., Cunha Â., Gomes N.C.M. (2016). Integrated Analysis of Bacterial and Microeukaryotic Communities from Differentially Active Mud Volcanoes in the Gulf of Cadiz. Sci. Rep..

[76] Li J., Yang J., Sun M., Su L., Wang H., Gao J., Bai S. (2020). Distribution and Succession of Microbial Communities Along the Dispersal Pathway of Hydrothermal Plumes on the Southwest Indian Ridge. Front. Mar. Sci..

[77] Li W., Dong X., Lu R., Zhou Y., Zheng P., Feng D., Wang Y. (2021). Microbial Ecology of Sulfur Cycling near the Sulfate–Methane Transition of Deep-sea Cold Seep Sediments. Environ. Microbiol..

[78] Bernardet J. (2015). Flavobacteriales Ord. Nov. Bergey’s Manual of Systematics of Archaea and Bacteria.

[79] Imhoff J.F. (2015). Ectothiorhodospiraceae. Bergey’s Manual of Systematics of Archaea and Bacteria.

[80] Le Moine Bauer S., Lu G.-S., Goulaouic S., Puzenat V., Schouw A., Barreyre T., Pawlowsky-Glahn V., Egozcue J.J., Martelat J.-E., Escartin J. (2023). Structure and Metabolic Potential of the Prokaryotic Communities from the Hydrothermal System of Paleochori Bay, Milos, Greece. Front. Microbiol..

[81] López-Pérez M., Haro-Moreno J.M., Iranzo J., Rodriguez-Valera F. (2020). Genomes of the “ *Candidatus* Actinomarinales” Order: Highly Streamlined Marine Epipelagic Actinobacteria. mSystems.

[82] Kato S., Hara K., Kasai H., Teramura T., Sunamura M., Ishibashi J.I., Kakegawa T., Yamanaka T., Kimura H., Marumo K. (2009). Spatial distribution, diversity and composition of bacterial communities in sub-seafloor fluids at a deep-sea hydrothermal field of the Suiyo Seamount. Deep Sea Res. Part I Oceanogr. Res. Pap..

[83] Liu X., Hu L., Lyu Q., Shao J. (2020). *Thiomicrorhabdus indica* sp. nov., an obligately chemolithoautotrophic, sulfur-oxidizing bacterium isolated from a deep-sea hydrothermal vent environment. Int. J. Syst. Evol. Microbiol..

