# Supplementary material for: Perturbations in Microbial Communities at Hydrothermal Vents of Panarea Island (Aeolian Islands, Italy)
Source: Biology (Basel). 2025 Jan 17;14(1):86. doi: 10.3390/biology14010086 (PMC11761264; doi:10.3390/biology14010086)
Supplement: Supplementary file 1 [file biology-14-00086-s001.zip › biology-3365727-supplementary.pdf]

Perturbations in Microbial Communities at Hydrothermal Vents of Panarea Island (Aeolian Islands, Italy)

Annamaria Gallo[1], Fabio Sposito[2], Manfredi Longo[2], Gianluca Lazzaro[2], Cinzia Giuseppina Caruso[2], Sabina Morici[2], Sergio Scirè Scappuzzo[2], Slobodanka Radovic[3], Valeria Villanova[1], Luca Vecchioni[1], Marco Arculeo[1], Rosa Alduina[1, 4]

[1]Dipartimento Scienze e Tecnologie Biologiche, Chimiche e Farmaceutiche, University of Palermo, Viale delle Scienze, 90128 Palermo, Italy

[2]Istituto Nazionale di Geofisica e Vulcanologia-sezione di Palermo, via Ugo La Malfa, 90146 Palermo, Italy

[3] IGA Technology Services, Via Linussio 51, 33100 Udine, Italy

[4]NBFC National Biodiversity Future Center, Piazza Marina 61, 90133 Palermo, Italy

Correspondence:

Rosa Alduina

valeria.alduina@unipa.it

Email:

annamaria.gallo01@unipa.it

marco.arculeo@unipa.it

valeria.villanova@unipa.it

luca.vecchioni@unipa.it

fabio.sposito@ingv.it;

manfredi.longo@ingv.it;

gianluca.lazzaro@ingv.it;

cinzia.caruso@ingv.it;

sabina.morici@ingv.it;

sergio.scire@ingv.it;

sradovic@igatechnology.com

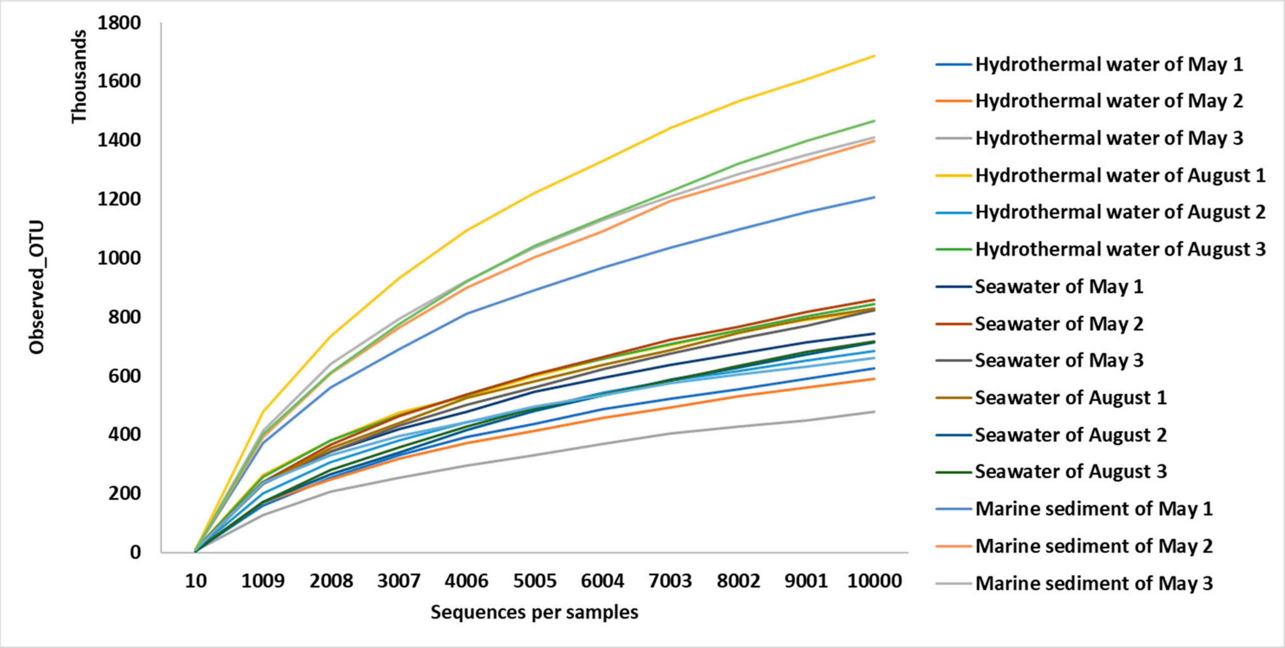

Figure S1 Rarefaction curves of the samples analysed in this study

Table S1. Total number of operational taxonomic units (OTUs) resulting from the pipeline dataset.

**Sample**	**OTU**
Hydrothermal water of May 1	29335
Hydrothermal water of May 2	20729
Hydrothermal water of May 3	17011
Hydrothermal water of August 1	19746
Hydrothermal water of August 2	21354
Hydrothermal water of August 3	26717
Seawater of May 1	27808
Seawater of May 2	29142
Seawater of May 3	20095
Seawater of August 1	35480
Seawater of August 2	62433
Seawater of August 3	50206
Marine sediment of May 1	20342
Marine sediment of May 2	36641
Marine sediment of May 3	32564

Marine sediment of August 1	37467
Marine sediment of August 2	34699
Marine sediment of August 3	45785

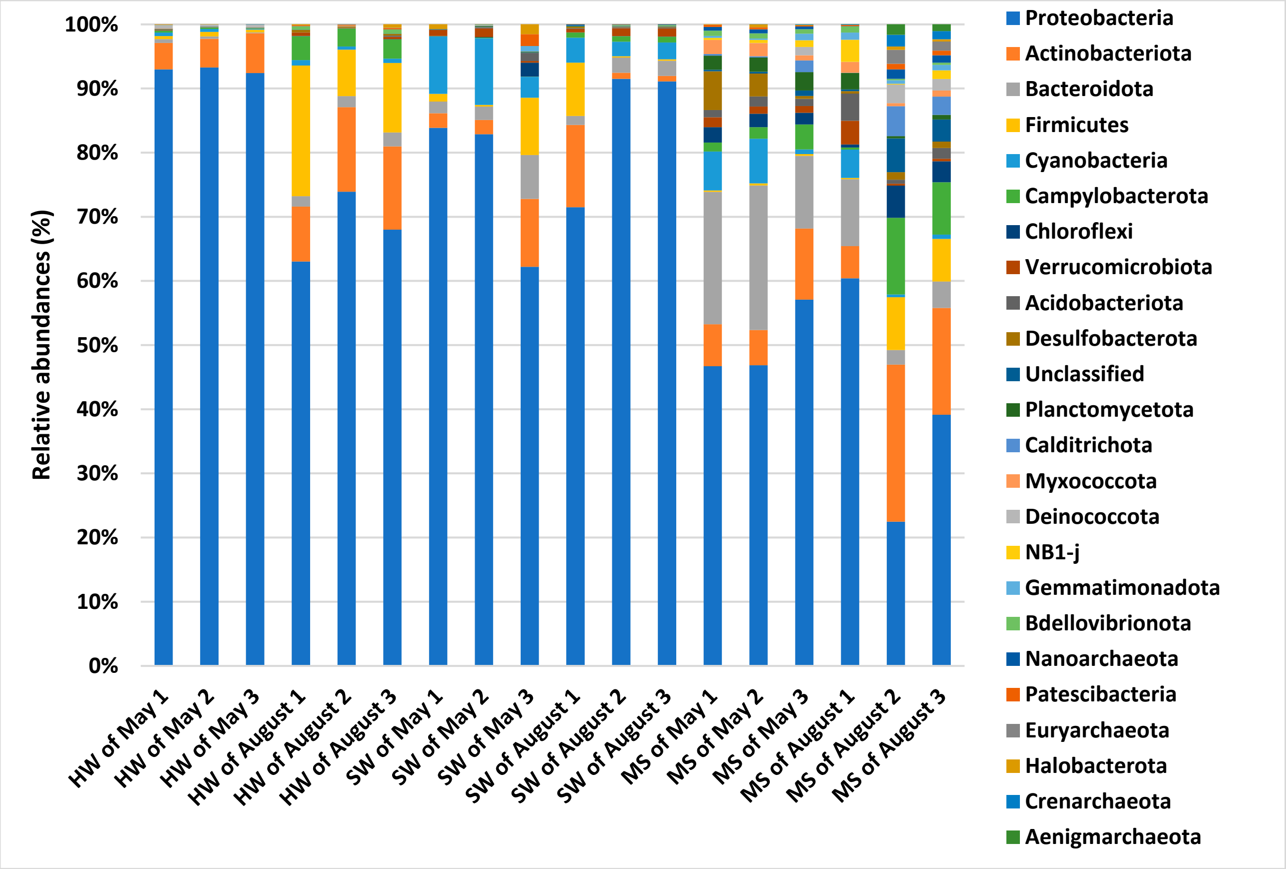

Figure S2 Percentage of relative abundance of the 25 main phyla found in all samples of this study

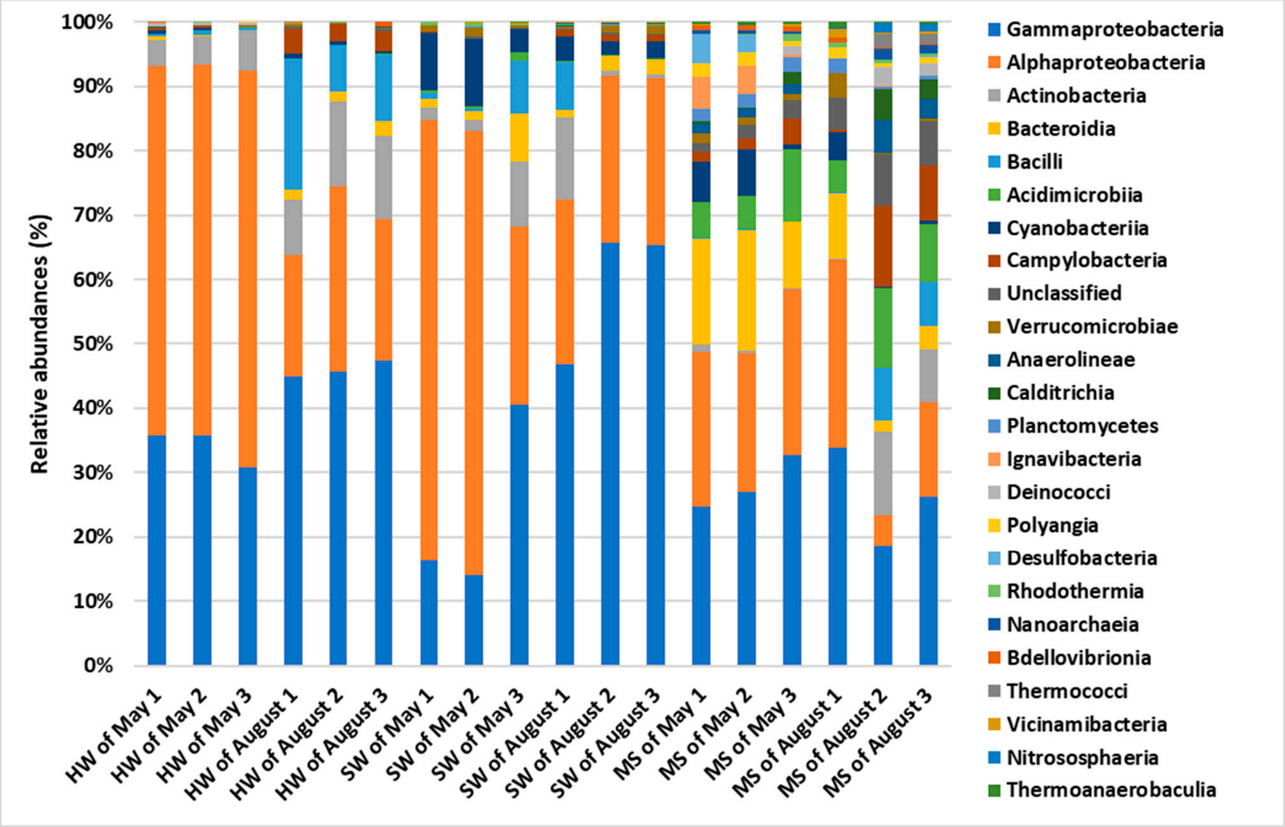

Figure S3 Percentage of relative abundance of the 25 main classes found in all samples of this study

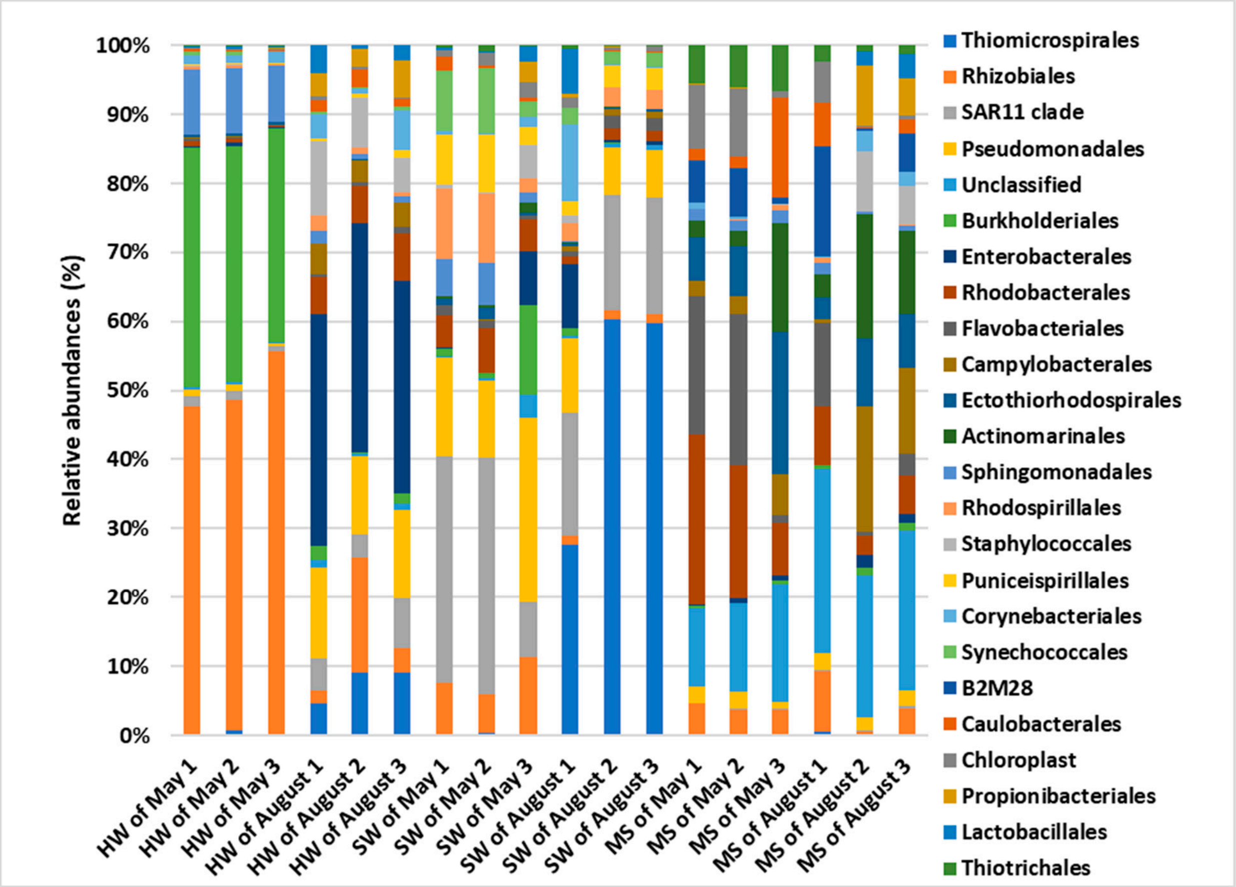

Figure S4 Percentage of relative abundance of the 25 main orders found in all samples of this study

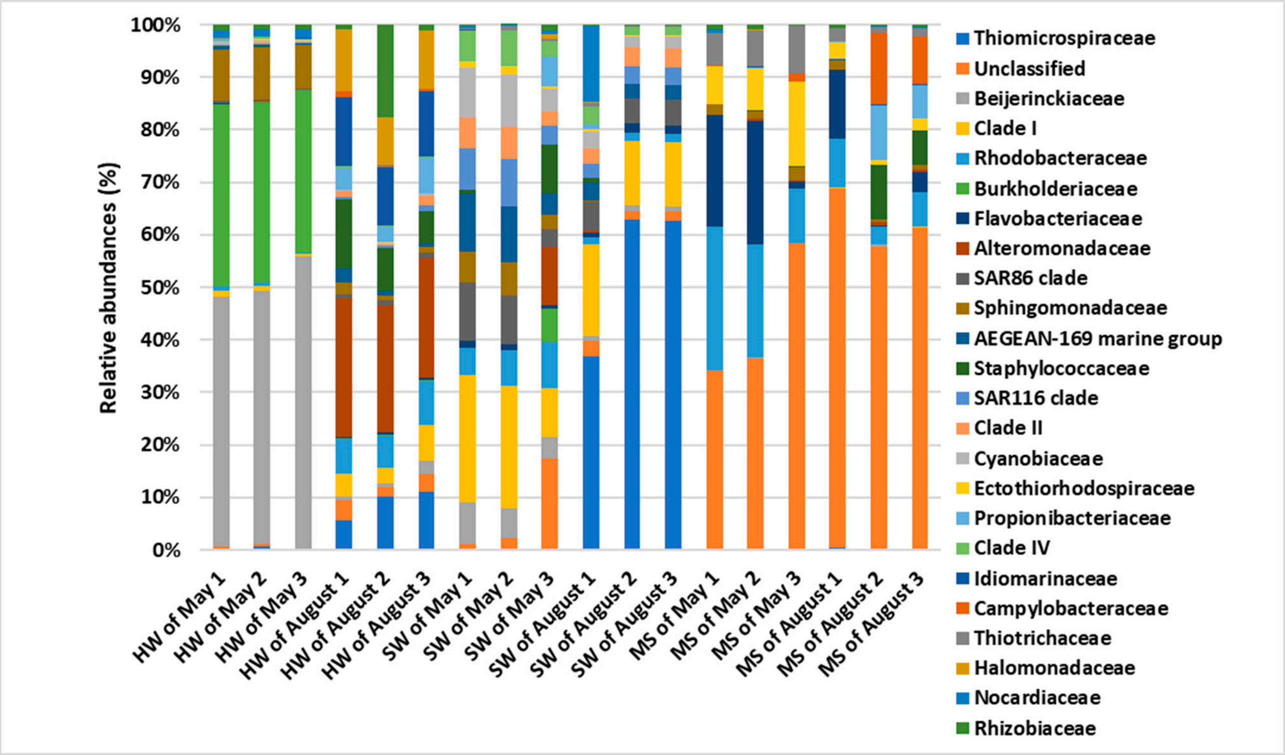

Figure S5 Percentage of relative abundance of the 25 main families found in all samples of this study

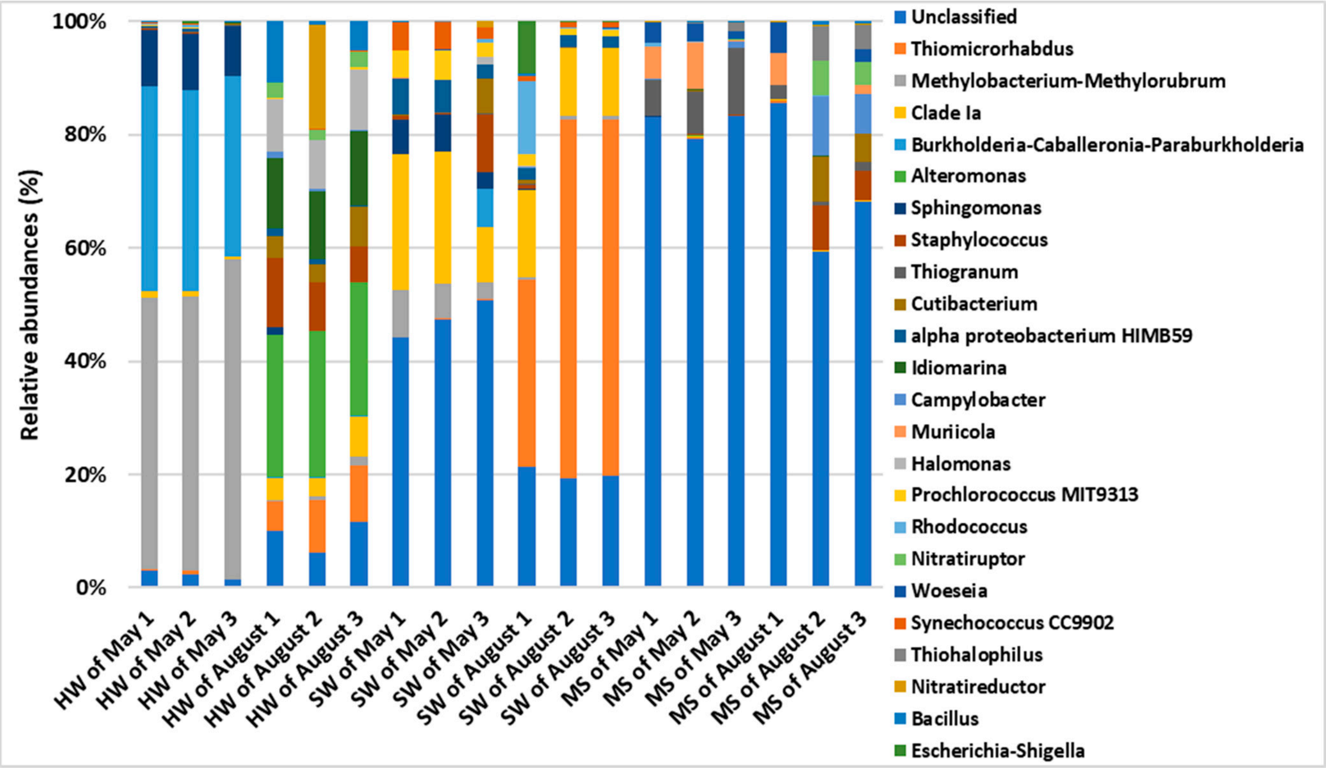

Figure S6 Percentage of relative abundance of the 25 main genera found in all samples of this study
